# Opinion: Mental health research: to augment or not to augment

**DOI:** 10.3389/fpsyt.2025.1539157

**Published:** 2025-02-18

**Authors:** Argyrios Perivolaris, Alice Rueda, Karisa Parkington, Achint Soni, Sirisha Rambhatla, Reza Samavi, Rakesh Jetly, Andrew Greenshaw, Yanbo Zhang, Bo Cao, Sri Krishnan, Venkat Bhat

**Affiliations:** ^1^ Interventional Psychiatry Program, St. Michael’s Hospital, Unity Health Toronto, Toronto, ON, Canada; ^2^ Cheriton School of Computer Science, University of Waterloo, Waterloo, ON, Canada; ^3^ Department of Management Science & Engineering, University of Waterloo, Waterloo, ON, Canada; ^4^ Vector Institute for Artificial Intelligence, Toronto, ON, Canada; ^5^ Institute of Mental Health Research, University of Ottawa, Ottawa, ON, Canada; ^6^ Department of Psychiatry, Faculty of Medicine and Dentistry, University of Alberta, Edmonton, AB, Canada; ^7^ Ministry of Health, Government of Alberta, Edmonton, AB, Canada; ^8^ Department of Computing Science, University of Alberta, Edmonton, AB, Canada; ^9^ Department of Electrical, Computer, and Biomedical Engineering, Toronto Metropolitan University, Toronto, ON, Canada; ^10^ Department of Psychiatry, University of Toronto, Toronto, ON, Canada

**Keywords:** augmentation, mental health, data scarcity, synthetic data, artificial intelligence

## Introduction

In recent years, there has been significant growth in the use of artificial intelligence (AI) and machine learning (ML) in healthcare. AI-based tools are increasingly used to predict diagnoses, personalize treatment plans, and assess risk factors, aiming to enable more scalable mental health care solutions. However, mental health research is often limited by the availability of high-quality and large sample datasets and confounded by the multifaceted complexities of human behaviors and emotions (1). To address this gap, researchers have begun to utilize data augmentation techniques to expand available datasets. Generating new data artificially enables models to use larger and more complete training datasets. Consider the medical imaging field, where AI has become a prominent fixture in practice. Data augmentation has demonstrated benefits across all organs and modalities to help promote medical imaging training without investing time and resources into collecting new samples (2). However, mental health research presents many unique barriers to integrating data augmentation. Biases inherent in the original set of mental health data remain and can result in overfitting where a model is unable to make accurate predictions from any other data other than the training data. This article explores the unique challenges researchers must overcome due to the lack of representative mental health data and how these challenges interact with AI and ML advancements. We explore data augmentation as a tool to bridge this gap, offering an integrative perspective on the ethical and practical challenges. As researchers consider data augmentation in mental health research, it is critical to evaluate the promise through rigorous methodologies and research and decide whether ‘to augment or not to augment’.

## What is data augmentation?

As AI and ML algorithms have advanced exponentially in recent years, one of the most prominent limiting factors remains the availability of representative training data that determines model performance (3). In contrast to synthetic data that creates data from scratch, data augmentation is an ML technique used to create new data based on existing data points, thereby artificially expanding a dataset. There are several data augmentation methods, with some incorporating simple transformations to text data (rotating images by random degrees, flipping images horizontally, and back-translation of data to a new language). Expanding on this, generative adversarial network (GAN) based augmentation is a more sophisticated strategy that uses neural networks to create novel samples from a pre-existing dataset. For example, GANs can augment data for chest X-rays that not only improve classification accuracy but perform better than other simple transformation methods (4). Large language models (LLMs), such as GPT-4o, have also been used for clinical transcript data augmentation (5). With various strategies available, data augmentation can be a potential tool for all fields of medical research moving forward. Augmentation can address class imbalance while preserving anonymity, facilitating cross-lingual and robust mental health research with available data. While data augmentation may enhance model generalizability and facilitate new research, mental health research introduces concerns about augmentation because of unique challenges in balancing realism and mitigating biases.

## To augment: overcoming data scarceness

Data scarcity remains a significant challenge in mental health research. Unlike other areas of medicine that can evaluate objective data from available biomarkers and imaging, mental health research relies on qualitative interviews, self-reported surveys, questionnaires, and clinical notes. The subjective nature of mental health concepts, such as emotional well-being (6), also makes developing universally accepted definitions challenging. Despite self-reported measurements being cost-efficient, flexible, and valuable for uncovering personal perceptions (7), many datasets do not provide the comprehensive, diverse, and sufficient data necessary for generalizable and reliable research. Furthermore, data collection is hindered by high costs, privacy concerns, stigma, and recruitment difficulties.

Augmented data presents a promising opportunity to address these issues. By artificially generating new data, such as augmented text or audio, researchers can increase usable data, mitigate the concerns of dependency on subjective reports of experiences, and enhance the scalability of mental health studies (8). Data augmentation is a cost-effective alternative to collecting new clinical data, reducing the reliance on expensive longitudinal studies. By using augmented data, researchers may limit the reliance on personally identifiable information, enhancing privacy protection. As well, researchers can instead focus efforts on generating new insights and testing hypotheses using readily available datasets.

Mental health datasets are often highly imbalanced, with certain conditions underrepresented (e.g., borderline personality disorder) compared to others (e.g., depression), and gender disparities in diagnosis, treatment, and research. Rare mental health disorders can present with uncommon symptoms, which can complicate diagnoses (9). Moreover, certain populations—such as children, seniors, racial minorities, LGBTQ2+, and marginalized groups—are also underrepresented in datasets. This imbalance can lead to biased conclusions and unreliable predictive models, which can perpetuate disparities and further marginalize underserved populations.

Addressing these issues, data augmentation can create more balanced datasets by artificially increasing the representation of minority classes, allowing ML models to better detect and treat underrepresented conditions and populations. AI-generated data can impute missing information and ensure datasets are more diverse, leading to more inclusive and equitable models. For instance, psychiatric symptoms often manifest differently across age groups and genders, with adolescents and adults experiencing distinct presentations of similar conditions (10). Augmented data allows for a better representation of subgroups, which can enhance diagnostic accuracy and treatment outcomes.

Augmentation is also crucial in scaling AI models. Introducing synthetic variations, such as noise injection, makes models more robust and less prone to overfitting. This increased variability enables models to learn general patterns rather than memorizing specific instances, thus improving their generalizability across different patient groups. This is particularly beneficial in mental health research, where there is significant variability in behavior and emotions. For example, consider a research team studying depressive disorders in a population skewed towards high symptom severity levels. An ML model trained on this real-world data may not predict accurate outcomes when applied to patient groups with lower symptom severity levels (11). However, researchers can achieve more accurate and generalizable predictions by generating augmented data that mimics these underrepresented cases (12). Incorporating data augmentation could improve research and clinical practice outcomes, allowing decision-support tools to be developed, and offering more equitable recommendations.

## Not to augment: bias and clinician fidelity

Mental health data is nuanced and profoundly contextual, with small variations in symptoms or patient perceptions potentially leading to different clinical outcomes. This type of data has multifactorial and complicated biological, psychological, and social components. One of the most significant risks of data augmentation is replicating and potentially amplifying biases present in the original datasets. If the original dataset underrepresents certain cultural, gender, or ethnic groups, these biases may be further embedded into the model. Poorly designed augmented data, if not inspected by mental health professionals, may fail to respect the nuanced interplay of different symptomatology and can mistakenly intensify biases present in the original dataset, which may also introduce new biases (13, 14). In mental health research, historical biases regarding race, gender, and socioeconomic status are well-documented and must be mitigated (15).

Creating augmented data may risk the loss of meaning, especially when nuanced cultural and individual differences are simplified. This could lead to generalized stereotypes or poor representation of complex mental health experiences (16). Augmented data may fail to consider the complexity of identities intersecting such factors, which may result in inaccurate predictions, leading to inconsistencies in treatment recommendations. This is especially problematic in mental health, where symptoms and coping mechanisms can vary greatly across cultures due to differences in language, values, and stigma around mental illness. Augmented data generated without consideration of cultural contexts might promote the development of AI models that misinterpret the mental health challenges of underrepresented populations. Traditional augmentation techniques may treat diverse groups as homogeneous, reducing cultural and ethnic variability to a few representative data points, thus risking generalization and misrepresentation.

Moreover, augmented data may lack clinical expertise and the ability to reproduce real-world patient behavior and presentation (17). Augmented data may oversimplify the variability that clinicians rely on for diagnosis and treatment. Adding synthetic noise or random data augmentation may alter key data features, causing a loss of context crucial to understanding mental health conditions. For example, AI-generated text transcripts of patient interviews might lack the subtle linguistic cues and emotional context necessary for a clinician’s judgment (18). This disconnect could result in models that appear highly accurate in theory but fail to translate into reliable real-world clinical support. Evaluating the quality of augmented data is particularly challenging in mental health due to the subjective nature of psychological assessments and a lack of consistent validation benchmarks.

## Discussion: harmonizing novelty with caution

Given the different perspectives in this argument, how should mental health research proceed with augmented data? The key is cautious optimism. While augmented data should not be dismissed outright, it must be integrated with real-world data in a way that preserves transparency and mitigates bias.

One approach is to utilize augmented data to supplement rather than replace real-world data. Combining both traditional and augmented datasets can enhance the dataset diversity without over-relying on synthetic information. Models trained on augmented data should also be evaluated by mental health professionals with standard accuracy metrics and qualitative appraisals. This will help ensure that augmented data-driven predictions align with clinical expertise and judgment. To integrate augmented data into mental health research effectively, researchers should prioritize pilot and feasibility studies to assess its practicality and ensure alignment with clinical expertise. Collaborative efforts based on these findings can also address challenges related to bias, equity, and implementation.

Societal and cultural norms heavily influence how mental health symptoms are expressed, understood, and treated. For example, some cultures may emphasize physical symptoms like headaches, while others focus on emotional or behavioural aspects (19). Data augmentation preserves the distributional properties of the original dataset, including those with small sample sizes, imbalances, or underrepresented features. By enhancing the diversity and representation within the dataset, models trained on well-augmented data are more likely to generalize effectively and exhibit reduced bias. Importantly, if cultural nuances are present in the original data but captured unevenly, data augmentation can help balance representation. This allows the model to better generalize across cultural subtleties, improving its fairness and applicability. Incorporating ethnographic insights or consulting cultural experts during data creation can further improve augmented data’s realism and applicability (20).

The financial implications of data augmentation are an important consideration in promoting global health equity. Researchers in wealthier regions often have greater access to the tools and funding, potentially exacerbating inequalities (21). In contrast, researchers in underserved areas may face significant barriers to adopting these technologies. Open Science initiatives could help promote the sharing of augmented datasets and tools, enabling broader access (21, 22). Publicly available platforms can democratize research opportunities, while transparency protocols requiring researchers to disclose their augmentation methods could foster collaboration and reduce disparities. By addressing these financial and equity concerns, the benefits of augmented data can be distributed more equitably across research communities (22).

Finally, the ethical implications of using augmented data in mental health research must not be overlooked. Augmented data can mitigate privacy concerns however, generating realistic patient data raises questions about consent and transparency. In addition, ethicists will need to develop clear guidelines for using augmented data in healthcare AI models that align with clinicians’ preferred practices and optimize patient confidentiality. Frameworks that promote positive clinician-AI interactions can ensure that AI data-driven models undertake the same rigorous inspection as models based on real-world data and be successfully implemented in clinical settings (23, 24).

## Conclusion

T he use of augmented data in mental health research is an exciting frontier, contributing to the potential to overcome long-standing challenges of data scarcity and imbalance. Data augmentation has been demonstrated to be a useful tool in other medical fields, such as medical imaging. However, introducing augmented data into the mental health field must be handled with caution. While the promise of enhanced model performance and data diversity is desirable, the risks of bias, unreliability, and ethical concerns may limit feasibility.
